# Beneficial Autophagic Activities, Mitochondrial Function, and Metabolic Phenotype Adaptations Promoted by High-Intensity Interval Training in a Rat Model

**DOI:** 10.3389/fphys.2018.00571

**Published:** 2018-05-23

**Authors:** Fang-Hui Li, Tao Li, Jing-Yi Ai, Lei Sun, Zhu Min, Rui Duan, Ling Zhu, Yan-ying Liu, Timon Cheng-Yi Liu

**Affiliations:** ^1^School of Sport Sciences, Nanjing Normal University, Nanjing, China; ^2^School of Physical Education and Health, Zhaoqing University, Zhaoqing, China; ^3^Laboratory of Laser Sports Medicine, South China Normal University, Guangzhou, China

**Keywords:** high-intensity interval training, continuous training, NMR spectroscopy, metabolomics, exercise tolerance

## Abstract

The effects of high-intensity interval (HIIT) and moderate-intensity continuous training (MICT) on basal autophagy and mitochondrial function in cardiac and skeletal muscle and plasma metabolic phenotypes have not been clearly characterized. Here, we investigated how 10-weeks HIIT and MICT differentially modify basal autophagy and mitochondrial markers in cardiac and skeletal muscle and conducted an untargeted metabolomics study with proton nuclear magnetic resonance (^1^H NMR) spectroscopy and multivariate statistical analysis of plasma metabolic phenotypes. Male Sprague–Dawley rats were separated into three groups: sedentary control (SED), MICT, and HIIT. Rats underwent evaluation of exercise performance, including exercise tolerance and grip strength, and blood lactate levels were measured immediately after an incremental exercise test. Plasma samples were analyzed by ^1^H NMR. The expression of autophagy and mitochondrial markers and autophagic flux (LC3II/LC3-I ratio) in cardiac, rectus femoris, and soleus muscle were analyzed by western blotting. Time to exhaustion and grip strength increased significantly following HIIT compared with that in both SED and MICT groups. Compared with those in the SED group, blood lactate level, and the expression of SDH, COX-IV, and SIRT3 significantly increased in rectus femoris and soleus muscle of both HIIT and MICT groups. Meanwhile, SDH and COX-IV content of cardiac muscle and COX-IV and SIRT3 content of rectus femoris and soleus muscle increased significantly following HIIT compared with that following MICT. The expression of LC3-II, ATG-3, and Beclin-1 and LC3II/LC3-I ratio were significantly increased only in soleus and cardiac muscle following HIIT. These data indicate that HIIT was more effective for improving physical performance and facilitating cardiac and skeletal muscle adaptations that increase mitochondrial function and basal autophagic activities. Moreover, ^1^H NMR spectroscopy and multivariate statistical analysis identified 11 metabolites in plasma, among which fine significantly and similarly changed after both HIIT and MICT, while BCAAs isoleucine, leucine, and valine and glutamine were changed only after HIIT. Together, these data indicate distinct differences in specific metabolites and autophagy and mitochondrial markers following HIIT vs. MICT and highlight the value of metabolomic analysis in providing more detailed insight into the metabolic adaptations to exercise training.

## Introduction

Emerging human and rodent model research data have shown greater cardiometabolic fitness and insulin sensitivity benefits from high-intensity interval training (HIIT), which is characterized by periods of high-intensity exercise combined with short rest intervals, compared with traditional moderate-intensity continuous training (MICT) (MacInnis et al., 2017). HIIT was reportedly superior to volume-matched MICT in enhancing insulin signaling in cardiac and skeletal muscle and reducing lipogenesis in adipose tissue of metabolic syndrome patients (Tjønna et al., 2008) and obesity-induced metabolic dysfunctions (Hafstad et al., 2013; Chavanelle et al., 2017), suggesting that HIIT may counteract metabolic dysregulation through altered substrate utilization and improved mechanoenergetics (Hafstad et al., 2013; Chavanelle et al., 2017; MacInnis et al., 2017). However, relatively few studies have investigated specific metabolic adaptations of HIIT or shown that HIIT is superior to MICT, matching total volume.

Metabolomics is an expanding area of systems biology, as metabolites represent functional end-products of gene expression that are closely associated with global phenotypic changes (Pechlivanis et al., 2010; Kuehnbaum et al., 2015). Proton nuclear magnetic resonance (^1^H NMR) spectroscopy-based non-targeted metabolomics represent a powerful technique for metabolome investigation in sport and exercise sciences (Pechlivanis et al., 2010). For investigating changes in metabolites between predefined states, principal component analysis (PCA) and partial least squares-discriminant analysis (PLS-DA) allow visualization of multidimensional relationships of measured variables (i.e., metabolites) to predefined states. To date, several studies have applied metabolomics to characterize metabolic signatures of exercise performance (Monleon et al., 2014; Overmyer et al., 2015; Falegan et al., 2017), including robust plasma metabolite profiles associated with moderate changes in the cardiorespiratory fitness status (Chorell et al., 2012) and the enhanced efficiency of fatty acid and branched-chain amino acid (BCAA) utilization in mitochondria (Overmyer et al., 2015). An early study by Peake et al. (2014) revealed distinct differences in specific metabolite responses to acute HIIT and energy-matched MICT. However, we are not aware of any studies that have systematically explored differences in metabolomic adaptations to HIIT relative to total volume-matched MICT.

More recent research has compared the molecular mechanism of mitochondrial metabolism adaptations between HIIT and MICT protocols (Granata et al., 2016). Moreover, improvement of mitochondrial function during exercise can be achieved by mitochondrial quality control, including biosynthesis of new mitochondria and concomitant removal of damaged mitochondria by autophagy, an essential cellular process that contributes to both the turnover of cellular components and damaged mitochondria as well as the response to exercise training. Moreover, defects in skeletal and cardiac muscle autophagy lead to mitochondrial carbohydrate (He et al., 2012), fat (Singh et al., 2009), and amino acid metabolism dysfunction. Conversely, the induction of skeletal and cardiac muscle autophagy during endurance training triggers beneficial adaptive changes in mitochondrial metabolism and is associated with enhanced physical fitness (He et al., 2012; Lira et al., 2013). Prior *in vivo* work has principally focused on the induction of autophagy in different organs, including the skeletal muscle and heart, during endurance training and a single bout of exercise (He et al., 2012). Few studies have compared basal autophagy and mitochondrial function in skeletal and cardiac muscle between long-term HIIT and total volume-matched MICT (Weng et al., 2013).

Thus, based on matched total exercise volume between two exercise protocols [i.e., HIIT exercise volume: four cycles (5 min × 18 m/min + 4 min × 42 m/min) + cool-down 28 m = MICT exercise volume: 34 min × 28 m/min + cool-down and warm-up 6 min × 18 m/min], the primary aim of this study was to compare the physical performance and molecular changes in skeletal and cardiac muscle and metabolomic patterns following 10-weeks HIIT and MICT in a rat model. To achieve this goal, we examined physical fitness by an exercise tolerance test, grip strength, and blood lactate level after the exercise tolerance test (Huang et al., 2016). The secondary aim was to investigate differences in basal autophagic and mitochondrial function markers in the cardiac and skeletal muscle between HIIT and MICT. Furthermore, we employed ^1^H NMR-based non-targeted metabolomic analysis to assess changes in carbohydrate, fat, and amino acid metabolism following 10- weeks HIIT and MICT programs.

## Methods

### Animal care

Six-week-old male Sprague–Dawley (SD) rats and standard laboratory chow were purchased from Guangdong Medical Laboratory Animal Center (GMLAC). The rats were kept on an artificial 12-h light-dark cycle (6:00 a.m.−6:00 p.m.) at room temperature (23±°C) in the Laboratory Animal Center, School of Sports Science and Physical Education, South China Normal University. Water and food were available *ad libitum*. The animals were housed in their respective groups in a collective cage and received water and standard laboratory chow. The experimental protocol was approved by the Ethics Committee on Animal Experimentation of the GMLAC and followed the Guidelines for the Care and Use of Laboratory Animals.

After 1 week of preconditioning feeding and 2 weeks of preconditioning running regimen, all rats were randomly assigned to three groups: sedentary control (SED, *n* = 10), MICT (*n* = 12), and HIIT (*n* = 12). After 48 h of rest following the final training, blood samples were collected into ice-cold EDTA capillary system tubes by the tail nick procedure, approximately 200 μl of blood plasma sample obtained after centrifugation was stored at −80°C until use for metabolomics analysis. After the exercise tolerance test, the rats were fasted for 24 h and sacrificed under carbon dioxide anesthesia. Subsequently, the soleus muscle, rectus femoris muscle, and left ventricular tissue were rapidly dissected in liquid nitrogen and frozen at −80°C until use for protein isolation and immunoblotting analysis.

### Exercise training protocols

The rats performed exercises based on a protocol described previously, with some modifications (Bedford et al., 1979). Briefly, before the exercise program, all animals underwent a preconditioning running regimen, which consisted of 10–30 min of daily running on a treadmill with a 10% grade at a speed of 10 m·min^−1^ during the first week and 30–40 min of daily running at 10 m·min^−1^ that was progressively increased by 2 m·min^−1^ until 20 m·min^−1^ during the second week. After the habituation period, maximal oxygen uptake (VO_2max_) was measured by means of expired gas analysis during a ramp protocol of a progressive exercise test, which consists on a treadmill exercise with 3 m/min increments every 3 min, and finishes when VO_2_ max is reached. VO_2_ max was defined as the VO_2_ after which an increase in work rate was not associated with a further increase (±5%) in continuously measured O_2_ uptake (Bedford et al., 1979). MICT included 3 min of warm-up at a constant running speed of 18 m·min^−1^, which corresponded to 35–40% of VO_2max_, followed by 34 min at a constant running speed of 28 m·min^−1^ (corresponding to 75–80% of VO_2max_) and cool-down at a constant running speed of 18 m·min^−1^ for 3 min. HIIT included 4 min of high-speed running at 42 m·min^−1^ at a 10% grade (four repetitions), which corresponded to 95–99% of VO_2max_, followed by 5 min of low-speed running at 18 m·min^−1^ (four repetitions) and cool-down at a constant running speed of 7 m·min^−1^ for 4 min. Therefore, the total volume for HIIT was 5.3 km·week^−1^, and the average intensity was about 75–80% of VO_2max_, which matched that of MICT. Both exercise modalities included five sessions per week for 10 weeks. An electrified grid (0.6-mA intensity) was placed behind the belt of the treadmill to induce running. SED group rats were age-matched rats that remained sedentary and were placed next to treadmills for an equivalent period during the training sessions.

### Physical fitness assessment

#### Exercise tolerance test

The exercise tolerance test consisted of walking at 12 m·min^−1^ for 3 min followed by 1.2 m·min^−1^ increases in speed every 2 min until the rat reached exhaustion. Time to exhaustion (s) was identified as the time until the rat sat at the lower end of the treadmill, near a shock bar, for 5 s (Huang et al., 2016).

#### Grip strength

Briefly, all rats were allowed to grasp the steel wire grid attached to the force gauge and were subsequently pulled back from the gauge. The force was recorded once they released the grid. Three trials were conducted, with the greatest force value recorded as the maximum grip strength and the force relative to body weight (BW) (N·g^−1^) using a grip strength meter (Bioseb, Shanghai, China) (Huang et al., 2016).

#### Blood lactate level after exercise tolerance test

It is known that higher serum lactate concentration is associated with intracellular acidification of skeletal muscle, which contributes to muscle fatigue, and is also a limiting factor of aerobic metabolism and used as an effective variable in determining muscle recovery after exercise (Huang et al., 2016). Blood lactate levels were determined prior to exercise (baseline), immediately after the exercise tolerance test, 10 min post-exercise tolerance test, and 3 h post-exercise tolerance test. Lactate Scout analyzer (EKF Diagnostics, Magdeburg, Germany) was employed to analyze 0.2 μl of blood obtained by tail nick (Neves et al., 2017).

### Protein analysis by immunoblotting

Left ventricular, soleus, and rectus femoris muscle was homogenized in tissue-lysing buffer. The supernatant from centrifugation was used for subsequent western blotting on a standard 10% SDS-PAGE gel in a Bio-Rad electrophoresis system (Hercules, CA, USA). The primary mitochondrial function was determined with antibodies to acetaldehyde dehydrogenase 2 (ALDH2, #ab108306), sirtuin 3 (SIRT3, #2627), cytochrome C oxidase subunit-IV (COX-IV, #4844), and succinate dehydrogenase (SDH, #11998). The primary autophagy activity was determined with antibodies to LC3A/B (#12741), ATG-3 (#3415), and Beclin 1 (#3495). Glyceraldehyde-3-phosphate dehydrogenase (GAPDH, #2118) as the loading control. All antibodies were purchased from Cell Signaling Technology (Danvers, MA, USA), except ALDH2, which was from Abcam (Cambridge, UK). Antibodies were diluted to 1:1,000, based on dilution curve optimization tests. Horseradish peroxidase-conjugated donkey anti-rabbit IgG (H+L; 711-035-152, Jackson ImmunoResearch Europe, Newmarket, UK) was used as the secondary antibody for 1 h at room temperature and washed with TBS-T. Bands were visualized used the Enhanced Chemiluminescence Detection Kit (Amersham Pharmacia Biotech, Little Chalfont, UK), and the approximate molecular weight of each protein was estimated using Precision Plus Protein Western C Standards and Precision Protein Strep-T Actin HRP Conjugate (BioRad). Chemiluminescence signal was captured using the ChemiDoc MP Imaging System (Bio-Rad, Gladesville, NSW, Australia), and digital images were generated. Resultant images were converted into a TIFF format and quantified using Image J software (NIH, Bethesda, MD, USA).

### Sample preparation and NMR spectroscopy

Plasma samples were vortexed for 30 s, and the aqueous layer was transferred to a 0.5-ml, 3-kDa ultrafiltration filter (Millipore, Burlington, MA, USA). Filtrate was collected by centrifuging the sample at 13,000 rpm for 45 min. The aqueous layer was transferred to a clean 2-ml centrifuge tube, and 450 μl D20 was added. Fifty microliters of DSS standard solution was added. Samples were mixed well before transfer to a 5-mm NMR tube (Norell, Morganton, NC, USA). Spectra were collected using an Agilent DD2-600 MHz spectrometer (Santa Clara, CA, USA) equipped with a triple-resonance cryoprobe. The first increment of a 2D-^1^H, ^1^H-NOESY pulse sequence was utilized for the acquisition of ^1^H-NMR data and suppression of the solvent signal. Experiments used a 100-ms mixing time along with a 990-ms pre-saturation (~80 Hz gamma B1). Spectra were collected at 25°C, with a total of 64 scans over a period of 7 min. Prior to Fourier transformation, an exponential line-broadening function of 0.5 Hz was applied to the free induction decay (FID). All plasma ^1^H NMR spectra were manually phased, baseline corrected, and referenced to DSS (δ 0.00) using Bruker Topspin 3.0 software (Bruker GmbH, Karlsruhe, Germany). Resonances were assigned according to literature and Chenomx NMR Suite (version 8.1, Chenomx, Edmonton, Canada). The aligned spectra over the range of δ 0.01–4.4 and δ 5.0–10.0 were binned into integrated segments of equal width of 0.04 ppm and normalized by probabilistic quotient normalization.

### Data processing and multivariate statistical analysis

Multivariate statistical analyses, including principal component analysis (PCA) and partial least squares-discriminant analysis (PLS-DA), were undertaken to analyze the spectral data. PCA is an unsupervised method that projects data points onto a plot for visualization of their distribution dependent on metabolite correlations that show the largest deviations across the data set, which can be utilized to examine trends within a data set without force fitting for differences between predefined groups. We carried out the data analysis in Metabo Analyst 3.0 software. PCA was applied to examine differentiation in the overall metabolic profile between SED, MICT, and HIIT groups. After that, PLS-DA was conducted to classify the groups' segregation and identify the most important metabolites that explain the changes in metabolic profile between groups. Metabolites with variable importance for projection (VIP) > 1.0 were classified as the most important metabolites in the segregation model (Falegan et al., 2017). Reduced PLS-DA models were tested with these metabolites, until a model with greater predictive capacity using the fewest possible variables was obtained. The robustness and quality of the models were reported by permutation tests (100 permutations) and cross-validation (R2Y and Q2). Univariate analyses were conducted to compare specific changes in each dependent variable arising from training between the SED and MICT or HIIT groups. The data distribution and homogeneity of variances were tested by the Shapiro-Wilk Test and Levene's test.

Values were presented as the mean ± standard deviation. Prior to statistical analysis, all data were checked for normality using the one-sample Kolmogorov-Smirnov test. Blood lactate levels were evaluated using two-way repeated measures analysis of variance (ANOVA). Comparisons of the metabolite intensities of each group were conducted by one-way ANOVA followed by Tukey's *post-hoc* test with false discovery rate (FDR) correction (Storey and Tibshirani, 2003). One-way ANOVA followed by Tukey's *post-hoc* test was used to analyze other measures. Cohen's d (effect sizes, ES) and associated confidence intervals (CI) were calculated to determine the magnitude of difference between the SED and MICT or HIIT groups for physical fitness and metabolite variables. These analyses were carried out using GraphPad Prism software version 6.0 (La Jolla, CA, USA). The level of statistical significance for all analyses was 95% (*P* < 0.05).

## Results

### Morphological characteristics

The morphological characteristics of rats in the three groups after the 10-weeks program are shown in Table 1. The final BW, perirenal adipose tissue weight, and relative perirenal adipose tissue weight were lower for the MICT and HIIT groups than for the SED group (*P* < 0.01). The relative quadriceps weight (*P* < 0.05), relative gastrocnemius weight (*P* < 0.05), and relative epididymis weight (*P* < 0.01) were higher for the MICT and HIIT groups than for the SED group. The quadriceps weight and gastrocnemius muscles weight were significantly increased in the HIIT group compared with those in the SED group (*P* < 0.05). However, the relative soleus weight and extensor digitorum longus muscle weight were comparable among the three groups (*P* > 0.05).

**Table 1:** Morphological characteristics of the experimental groups.

	**SED (*n* = 10)**	**MICT (*n* = 12)**	**HIIT (*n* = 12)**
Initial BW (g)	225 ± 4.0	224 ± 6.0	222 ± 3.0g
Final BW (g)	674 ± 45	525 ± 24^**^	520 ± 38^**^
Perirenal adipose tissue weight (g)	17.0 ± 3.20	5.73 ± 2.80^**^	5.04 ± 1.85^**^
Quadriceps weight (g)	10.4 ± 1.10	9.25 ± 0.28	8.78 ± 0.68^*^
Gastrocnemius weight (g)	7.76 ± 0.84	6.95 ± 0.45	6.27 ± 0.63^*^
Soleus muscles weight (g)	0.65 ± 0.22	0.57 ± 0.08	0.61 ± 0.14
Extensor digitorum longus weight (g)	0.68 ± 0.16	0.58 ± 0.08	0.60 ± 0.05
Epididymis weight (g)	4.10 ± 0.18	3.76 ± 0.17	3.75 ± 0.38
Relative perirenal adipose tissue weight (%)	2.60 ± 0.50	1.10 ± 0.50^**^	1.00 ± 0.40^**^
Relative quadriceps weight (%)	1.61 ± 0.14	1.81 ± 0.13^*^	1.78 ± 0.09^*^
Relative gastrocnemius weight (%)	1.16 ± 0.09	1.41 ± 0.09^*^	1.32 ± 0.08^*^
Relative soleus muscles weight (%)	0.10 ± 0.03	0.11 ± 0.02	0.13 ± 0.03
Relative extensor digitorum longus weight (%)	0.10 ± 0.03	0.12 ± 0.01	0.12 ± 0.01
Relative epididymis weight (%)	0.62 ± 0.02	0.75 ± 0.03^**^	0.76 ± 0.06^**^

*Values are reported as the mean ± standard deviation. Groups: SED, sedentary control; MICT, moderate-intensity continuous training; HIIT, high-intensity interval training. BW, body weight; Relative tissue weight (%) = tissue weight (g)/final body weight (g) × 100%*.

* *P < 0.05*,

** *P < 0.01. Means with different superscripts in each row are significantly different from SED (one-way ANOVA followed by Tukey's post-hoc test)*.

### Time to exhaustion, grip strength, and blood lactate level after an incremental exercise test

The mean running time to exhaustion, grip strength, and blood lactate levels from the rats in the three groups after 10 weeks of exercise training are shown in Table 2. One-way ANOVA suggested that time to exhaustion in both exercise modalities was significantly increased compared with that of the SED group (MICT vs. SED: ES = 2.16; HIIT vs. SED: ES = 3.87; *P* < 0.01). Significant differences in grip strength (HIIT vs. SED: ES = 2.12; *P* < 0.01) between the HIIT and SED groups were found. Time to exhaustion (HIIT vs. MICT: ES = 1.36; *P* < 0.05) and grip strength (HIIT vs. MICT: ES = 1.30; *P* < 0.05) were significantly increased in the HIIT group compared with those in the MICT group.

**Table 2:** Effect sizes with confidence intervals for time to exhaustion, grip strength, and blood lactate level.

	**SED (*n* = 10)**	**MICT (*n* = 12)**	**HIIT (*n* = 12)**	**MICT vs. SED**		**HIIT vs. SED**		**HIIT vs. MICT**	
				**%changed ES (CI)**		**%changed ES (CI)**		**%changed ES (CI)**	
**Time to exhaustion (min)**	35.6 ± 14.0	83.6 ± 28.7^**^	120.8 ± 28.4^**^^#^	135	2.16 (−14 to 10.8)	239	3.87 (−12.2 to 12.6)	44.5	1.36 (−14.7 to 19.2)
**Grip strength (N**·**g**^−1^**)**	3.64 ± 0.60	4.37 ± 0.37	5.31 ± 0.97^**^^#^	20.1	1.57 (1.4 to 1.9)	45.9	2.12 (1.4 to 1.9)	21.5	1.30 (1.6 to 2.5)
**Blood lactate level (mol**·**l**^−1^**)**									
Pre-EX	1.43 ± 0.21	1.38 ± 0.20	1.45 ± 0.25	−3.50	−0.3 (−0.4 to 0.1)	1.40	0.09 (−0.01 to 0.23)	5.07	0.32 (−0.01 to 0.23)
I-EX	6.48 ± 3.40	3.28 ± 0.72	2.81 ± 0.60	−49.4	−1.43 (−1.8 to 0.7)	−56.6	−1.66 (−2.0 to 0.5)	−14.3	−0.74 (−1.1 to −0.3)
10 minPOST	3.28 ± 2.40	2.11 ± 0.41	1.65 ± 0.75^*^	−35.7	−0.75 (−1.0 to 0.7)	−49.7	−1.00 (−1.4 to 0.5)	−21.8	−0.78 (−1.2 to −0.5)
Exercise-time interaction	*P* = 0.0002								
Main effect for time	*P* < 0.0001								
Main effect for exercise	*P* = 0.02								

Values are mean ± standard deviation. ES, effect size; CI, confidence intervals. Groups: SED, sedentary control; MICT, moderate-intensity continuous training; HIIT, high-intensity interval training. I-EX, blood lactate level immediately after an incremental exercise test; 10 minPOST, blood lactate level 10 min after an incremental exercise test;

* Significantly different from SED group rats (P < 0.05);

** *Significantly different from SED group rats (P < 0.01)*;

# *Significantly different from MICT group rats (P < 0.01)*.

Two-way ANOVA revealed that the two exercise modalities decreased blood lactate levels after running to exhaustion. A significant two-way interaction (exercise-time interaction; *P* = 0.0002), main effect for time (*P* < 0.0001), and main effect for exercise (*P* = 0.02) for blood lactate levels were observed (Table 2). Significant differences in blood lactate levels immediately after an incremental exercise test (I-EX) (ES = −1.66; *P* < 0.01) and 10 min after an incremental exercise test (10 minPOST) (ES = −1.0; *P* < 0.05) between the HIIT and SED groups were identified. Significant differences in blood lactate levels at I-EX (MICT vs. SED: ES = −1.43; *P* < 0.01) between the MICT and SED groups were also noted.

### Protein expression of autophagy and mitochondrial function markers

**Figure 1:**
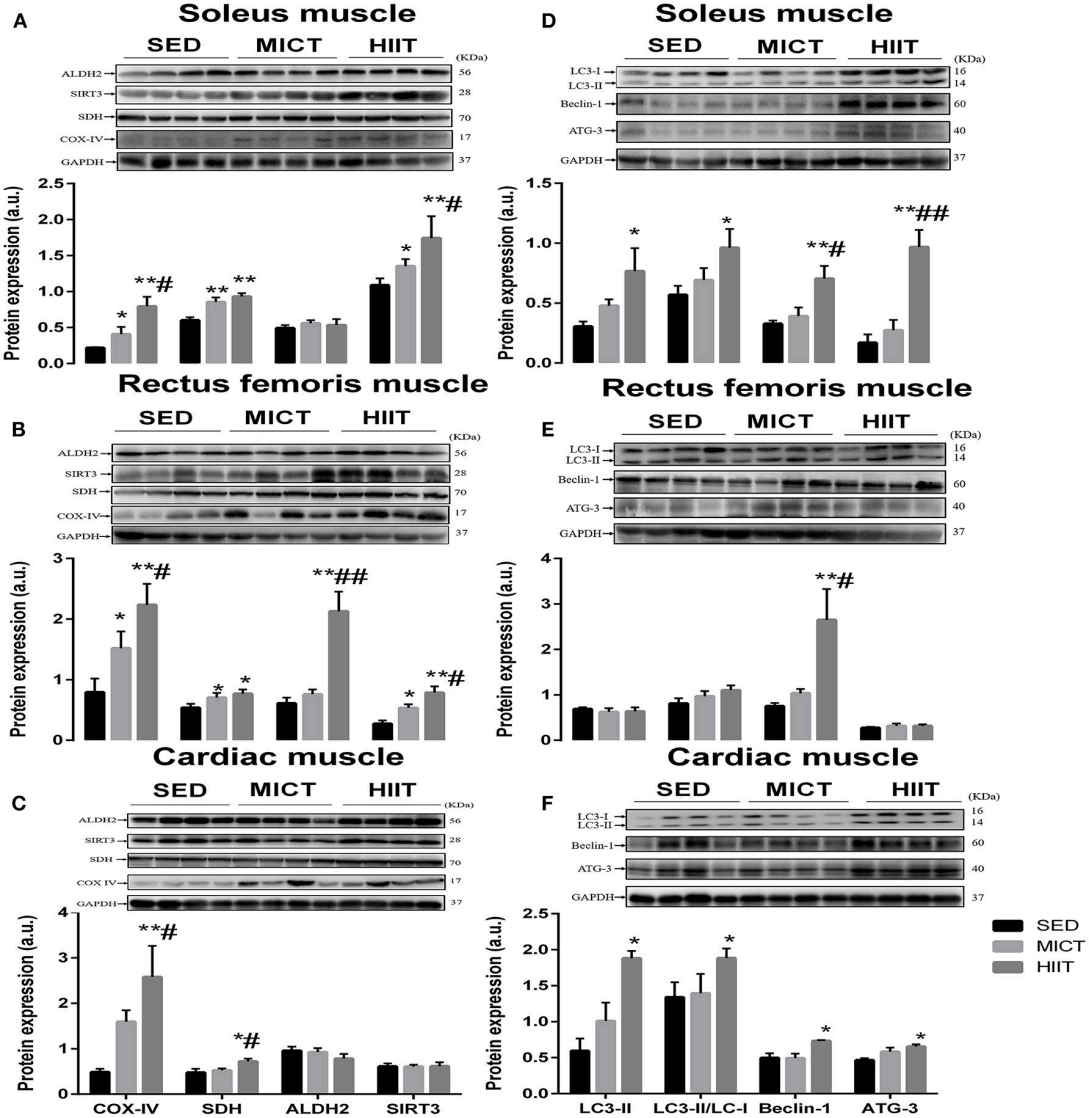
Protein content of autophagy and mitochondrial markers in soleus, rectus femoris, and cardiac muscle. Changes in content of mitochondrial function and autophagy markers in soleus **(A,D)**, rectus femoris **(B,E)**, cardiac muscle **(C,F)** in the three experimental groups. Levels of detected proteins were normalized to GAPDH. Densitometry analysis was performed to quantify the expression levels of detected proteins (a. u.). Values are mean ± standard deviation (*n* = 4). Groups: SED, sedentary control; MICT, moderate-intensity continuous training; HIIT, high-intensity interval training; ^*^Significantly different from SED group rats (*P* < 0.05); ^**^Significantly different from SED group rats (*P* < 0.01); ^#^ Significantly different from MICT group rats (*P* < 0.05); ^##^ Significantly different from MICT group rats (*P* < 0.01).

We compared mitochondrial function by measuring the expression of SDH, COX-IV, SIRT3, and ALDH2 proteins from rectus femoris, soleus, and left ventricular muscle that were harvested from HIIT, MICT, and SED group rats by western blotting (Figure 1). Both exercise modalities significantly elevated the expression level of COX-IV and SDH in the rectus femoris and soleus muscle compared with that in SED group rats (Figures 1A,B); meanwhile, SDH and COX-IV expression was upregulated significantly in left ventricular muscle from HIIT compared with that in SED group rats (Figure 1C). Additionally, a significant difference (*P* < 0.05) was seen in the COX-IV content in these three muscles and the SDH content of left ventricular muscle from HIIT compared with those in the MICT group (Figures 1A–C). The content of SIRT3 significantly increased in the soleus (Figure 1A) and rectus femoris muscle (Figure 1B) in the HIIT group relative to that of the SED (*P* < 0.01) and MICT groups (*P* < 0.05), while HIIT significantly (*P* < 0.01) elevated ALDH2 content in the rectus femoris muscle compared with that in SED and MICT group rats (Figure 1B).

We next assessed the autophagy markers LC3-II, ATG-3, Beclin-1, and LC3II/LC3-I ratio (Figures 1D–F) in these muscles. In the left ventricular and soleus muscle (Figures 1D,F), HIIT significantly elevated all autophagy markers in comparison with those in the SED group. Additionally, a significant difference was seen in the ATG-3 (*P* < 0.01) and Beclin-1 (*P* < 0.05) content of soleus muscle (Figure 1D) and Beclin-1 (*P* < 0.05) content of rectus femoris muscle (Figure 1E) in the HIIT group compared with that in the MICT group.

### Correlation between autophagy and mitochondrial markers

Interaction analysis between autophagy and mitochondrial markers in the left ventricular and skeletal muscle is presented in Table 3. In the left ventricular muscle, a positive correlation between LC3-II and LC3-II/LC3-I ratio (*r* = 0.75), ATG-3 (*r* = 0.76), Beclin-1 (*r* = 0.67), SDH (*r* = 0.68), and COX-IV (*r* = 0.60) was observed; meanwhile, similar associations were found between LC3-II/LC3-I ratio and ATG-3 (*r* = 0.72), Beclin-1 (*r* = 0.66), SDH (*r* = 0.65), and COX-IV (*r* = 0.65); ATG-3 content was positively correlated with SDH (*r* = 0.63), COX-IV (*r* = 0.65), and Beclin-1 (*r* = 0.75). A positive correlation between ALDH2 content and COX-IV (*r* = 0.71) and SIRT3 (*r* = 0.71) was discovered as well as a positive correlation between COX-IV and Beclin-1 (*r* = 0.59).

**Table 3:** Correlations between autophagy or mitochondrial markers in cardiac and skeletal muscle.

	**Cardiac muscle (C.M)**							**Soleus muscles (S.M)**							**Rectus femoris muscles (R.M)**						
	**Autophagy**			**Mitochondrial**				**Autophagy**			**Mitochondrial**				**Autophagy**			**Mitochondrial**			
	**LC3-II**	**LC3-II/I**	**ATG-3**	**Beclin1**	**SDH**	**COX-IV**	**SIRT3**	**LC3-II**	**LC3-II/I**	**ATG-3**	**Beclin1**	**SDH**	**COX-IV**	**SIRT3**	**LC3-II**	**LC3-II/I**	**ATG-3**	**Beclin1**	**SDH**	**COX-IV**	**SIRT3**
**C.M**																					
LC3-II																					
LC3-II/I	**0.75**																				
ATG-3	**0.76**	**0.72**																			
Beclin1	**0.67**	**0.66**	**0.75**																		
SDH	**0.68**	**0.65**	**0.63**	0.48																	
COX-IV	**0.60**	**0.65**	**0.65**	**0.59**	0.31																
SIRT3	0.30	0.04	0.17	0.38	0.24	−0.2															
ALDH2	0.01	0.13	0.11	0.18	0.01	**0.71**	**0.71**														
**S.M**																					
LC3-II/I								0.42													
ATG-3								0.48	−0.1												
Beclin1								**0.73**	0.02	**0.72**											
SDH								**0.67**	**0.58**	**0.74**	**0.64**										
COX-IV								**0.75**	0.08	**0.82**	**0.78**	**0.76**									
SIRT3								0.19	**0.61**	0.08	0.12	**0.58**	**0.72**								
ALDH2								−0.1	0.01	−0.1	−0.43	−0.1	−0.1	0.01							
**R.M**																					
LC3-II/I															−0.1						
ATG-3															0.26	0.49					
Beclin1															−0.2	0.21	0.02				
SDH															−0.1	0.37	0.31	0.15			
COX-IV															−0.1	0.13	−0.13	**0.72**	**0.60**		
SIRT3															−0.3	0.46	0.01	**0.66**	**0.74**	**0.87**	
ALDH2															−0.1	0.32	0.22	**0.94**	0.25	**0.66**	**0.63**

*Values are Pearson correlation coefficient (r). Pearson correlations were calculated for the above analyses (n = 12, pooled samples of SC, MICT, and HIIT groups, n = 4 per group); Black bold represents significant difference (P < 0.05) for Pearson correlation. R.M, rectus femoris muscle; S.M, soleus muscle; C.M, cardiac muscle*.

In the soleus muscle, a positive correlation between LC3-II and Beclin-1 (*r* = 0.73), SDH (*r* = 0.67), and COX-IV (*r* = 0.75) was observed; similar associations were found between ATG-3 and Beclin-1 (*r* = 0.72), SDH (*r* = 0.74), and COX-IV (*r* = 0.82); Beclin-1 content was positively correlated with SDH (*r* = 0.64) and COX-IV (*r* = 0.78), and a positive correlation between COX-IV and SDH was observed (*r* = 0.76). Meanwhile, a positive correlation between SIRT3 and SDH (*r* = 0.58), LC3-II/LC3-I ratio (*r* = 0.61), and COX-IV (*r* = 0.72) was discovered as well as a positive correlation between SDH and LC3-II/LC3-I ratio (*r* = 0.58).

In the rectus femoris muscle, a positive correlation between SIRT3 and Beclin-1 (*r* = 0.66), SDH (*r* = 0.74), and COX-IV (*r* = 0.87) was discovered as well as a positive correlation between ALDH2 and COX-IV (*r* = 0.66), Beclin-1 (*r* = 0.94), and SIRT3 (*r* = 0.63).

### Multivariate statistical analysis of metabolites

**Figure 2:**
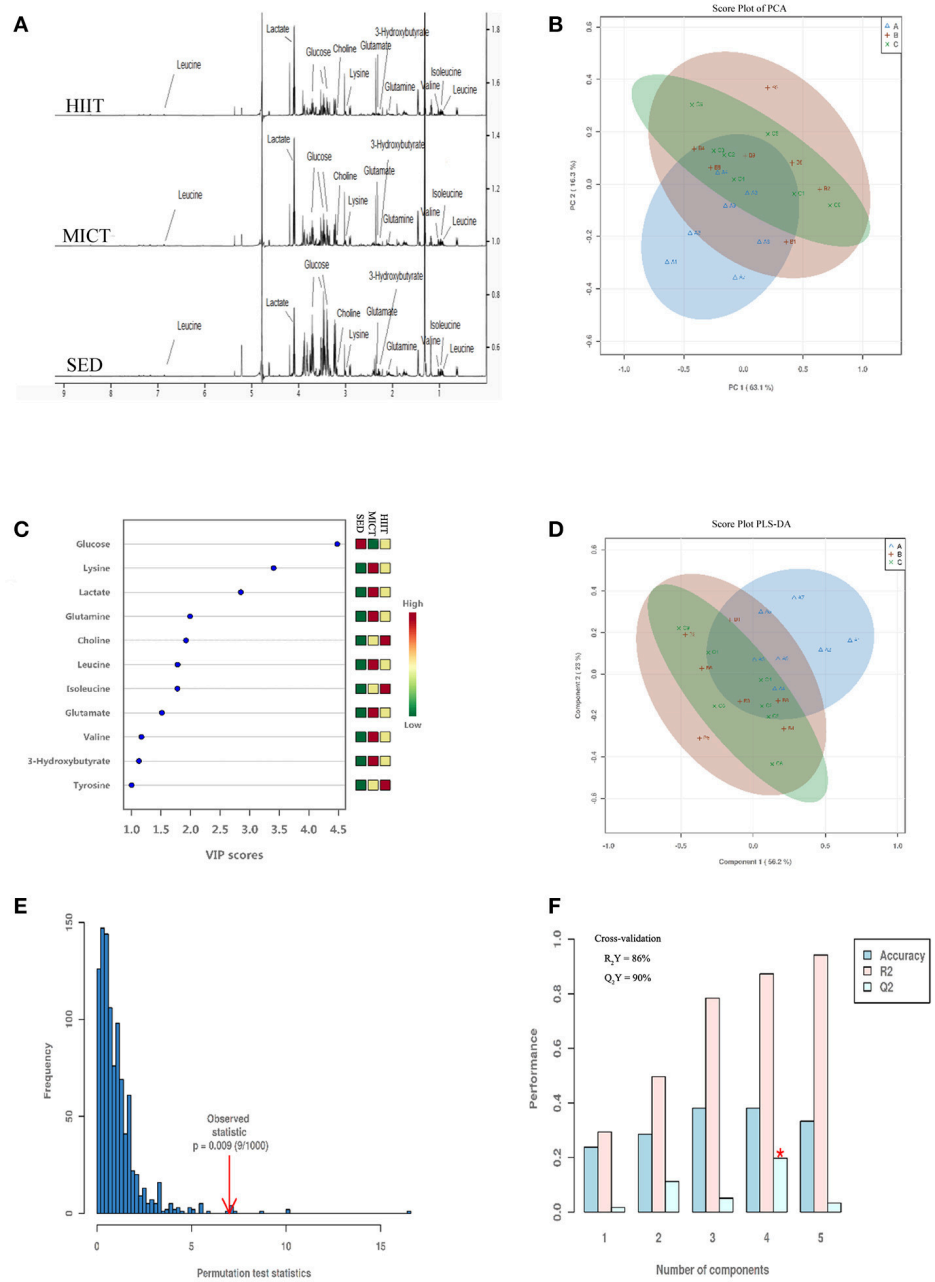
Representative 600-MHz NMR spectra, score plot of principal component analysis (PCA), and model of partial least squares-discriminant analysis (PLS-DA) of all serum samples. NMR spectra **(A)**, score plot of PCA **(B)** and VIP score **(C)**, score plot of PLS-DA **(D)**, the 100 permutations test **(E)**, and cross-validation score plots of PLS-DA **(F)**. Sedentary control (SED) is represented in blue Δ A; moderate-intensity continuous training (MICT) in red+B; high-intensity interval training (HIIT) in green × C in **(B,D)**. On the right in **(C)**, the levels (high-low) of metabolites in SED, MICT, and HIIT groups (*n* = 10) are represented.

H^1^ NMR spectroscopy and PCA and PLS-DA of serum from the three treatment groups are shown in Figure 2. A total of 38 metabolites were identified by Chenomx software (Figure 2A). In Figure 2B, the PCA model shows high segregation between groups HIIT or MICT and SED. As shown in the score plot of the PCA model in Figure 2B, the X- and Y-axes indicate the first component (PC1, 63.1%) and second component (PC2, 16.3%), respectively, and one dot represents one observation sample. The distances between dots represent the similarity of the samples' metabolite composition.

Supervised PLS-DA is a method that isolates the metabolites with the largest variation between predefined groups, allowing individual metabolites to be isolated by sensitivity and selectivity of group prediction, which allows a refocus of analysis to understand differences related specifically to the experimental question, thereby reducing the impact of systemic variation that may influence PCA models. High segregation between groups HIIT or MICT and SED is demonstrated, while low segregation in the samples for both HIIT and MICT groups is also shown in Figure 2D. Figure 2C presents the 11 metabolites with VIP score > 1.0 (glucose, lysine, lactate, glutamine, choline, leucine, isoleucine, glutamate, valine, 3-hydroxybutyrate, tyrosine). They were classified as the most important metabolites in the segregation model. The four principal-component model explained 86% (R2Y) and predicted 90.0% (Q2Y) of the data according to the cross-validation (Figure 2F), and the robustness of this model was measured by 100 permutations with *P* = 0.009 (Figure 2E). Thus, the model was very good for discriminating and separately clustering these samples.

### Changes in metabolite levels

Comparison of the data by one-way ANOVA followed by Tukey's *post-hoc* test with false discovery rate (FDR) correction between metabolites among groups, %change, and Cohen's d ES between groups are presented in Table 4. Glucose showed a differential decrease from both exercise modalities compared with that of the SED group. Lysine, choline, 3-hydroxybutyrate, and tyrosine showed a differential increase in both exercise modalities compared with those of the SED group, while BCAAs isoleucine, leucine, valine, and glutamine presented a significant increase in the HIIT group compared with in the SED group. However, there were no differences in the 11 metabolites between MICT and HIIT groups (Table 4).

**Table 4:** One-way ANOVA followed by Tukey's *post-hoc* test with FDR correction and effect sizes for metabolites relative to SED after HIIT and MICT protocols.

**Metabolite**	**Shift (ppm)**	**SED (*n* = 10)**	**MICT (*n* = 12)**	**HIIT (*n* = 12)**	**MICT vs. SED**			**HIIT vs. SED**			**HIIT vs. MICT**		
					**%changed**	**P**	**ES**	**%changed**	**P**	**ES**	**%changed**	**P**	**ES**
Glucose	3.22	0.052 ± 0.004	0.046 ± 0.004	0.047 ± 0.005	−13	**0.002**	−1.57	−13	**0.024**	•1.15	1.00	1.058	0.23
Lactate	4.1	0.074 ± 0.015	0.091 ± 00.027	0.086 ± 00.032	23	0.164	0.80	16	0.383	0.49	0.95	2.790	−0.18
Lysine	2.98	0.004 ± 0.0008	0.0053 ± 0.0006	0.0052 ± 0.0006	32	**0.022**	**1.96**	31	**0.004**	1.81	0.99	1.254	−0.15
3–hydroxybutyrate	2.26	0.0058 ± 0.0006	0.0077 ± 0.001	0.0078 ± 0.002	33	**0.036**	**2.36**	35	**0.029**	1.36	0.89	1.845	0.06
Choline	3.18	0.012 ± 0.002	0.0154 ± 00.002	0.0155 ± 0.002	28	**0.050**	**1.78**	29	**0.033**	1.84	1.01	0.922	0.05
Glutamine	2.42	0.015 ± 00.002	0.0186 ± 00.004	0.0186 ± 00.003	24	0.063	1.16	24	**0.027**	1.46	1.00	0.988	0
Glutamate	2.34	0.015 ± 0.003	0.0192 ± 0.007	0.0182 ± 0.006	28	0.166	0.79	21	0.271	0.69	0.95	2.163	−0.16
Isoleucine	0.98	0.012 ± 00.003	0.015 ± 00.004	0.016 ± 00.002	25	0.098	0.004	35	**0.030**	1.68	0.95	3.944	0.34
Leucine	0.94	0.016 ± 00.003	0.0194 ± 0.004	0.0206 ± 0.002	21	0.114	0.99	29	**0.032**	1.93	0.99	1.402	0.41
Valine	3.58	0.0065 ± 00.001	0.0079 ± 00.001	0.0082 ± 00.001	22	0.161	1.47	26	**0.027**	1.78	1.10	3.166	0.31
Tyrosine	6.86	0.0015 ± 0.0002	0.0021 ± 0.0003	0.0022 ± 00.0002	43	0.021	3.15	45	**0.001**	3.67	1.01	1.625	0.52

*Values are mean ± standard deviation. Comparisons of the metabolite intensities of each group were performed by one-way ANOVA followed by Tukey's post-hoc test with false discovery rate (FDR) correction to avoid false positives, and a value of P < 0.05 was considered statistically significant. Groups: SED, sedentary control; MICT, moderate-intensity continuous training; HIIT, high-intensity interval training. ES, effect size; Black bold represents significant difference between both exercise modalities and SED group; Red bold represents significant difference between HIIT and SED groups*.

## Discussion

This study revealed differential effects of HIIT and MICT on physical fitness, mitochondrial biogenesis marker proteins, basal autophagic activity, and metabolomics. The major findings were as follows: when compared with that in the SED and MICT groups, the HIIT protocol resulted in a larger improvement in physical fitness as assessed by grip strength and time to exhaustion; mitochondrial biogenesis markers were markedly higher in the skeletal and left ventricular muscle after HIIT compared with those after MICT; autophagy markers were significantly increased in the soleus and left ventricular muscle in the HIIT but not MICT group, when compared with those of the SED group. Furthermore, a metabolomics strategy showed that both HIIT and MICT programs promoted an increase in lysine, choline, 3-hydroxybutyrate, and tyrosine, but BCAAs and glutamine were specifically upregulated by HIIT when compared with those of the SED group. Collectively, these data indicated distinct differences in specific metabolites and autophagy and mitochondrial markers following HIIT vs. MICT and highlight the value of metabolomic analysis, which provides more detailed insight into the metabolic adaptation to long-term exercise training.

### Mitochondrial function

Increases in the mitochondrial biogenesis and oxidative phosphorylation capacity of skeletal muscle are reportedly associated with improvements in exercise tolerance (MacInnis et al., 2017). In the current study, 10-weeks HIIT and MICT programs did alter the expression of mitochondrial biogenetic markers SDH and COX-IV in the skeletal relative to those in the SED group (Figures 1A,B), which is consistent with the results of significantly greater increases in time to exhaustion as well as lower blood lactate level (a limiting factor of aerobic metabolism) immediately after the incremental exercise test to exhaustion (Table 2), suggesting that elevated mitochondrial oxidative capacity is involved in improved aerobic capacity following both exercise modalities. This is consistent with recent data demonstrating the positive effects of both exercise modalities on SDH activities in the soleus muscles compared with those in the SED group (Criswell et al., 1993; Edgett et al., 2016). These data were in line with previous work in a rodent model of hypertension, which showed that both exercise modalities increased COX-IV and SDH content in red gastrocnemius (Holloway et al., 2015).

Notably, our data showed that COX-IV content among three tissues (Figures 1A–C) and SDH content of left ventricular muscle increased more after HIIT than after matched-volume MICT (Figure 1C), suggesting that HIIT was superior to MICT in enhancing expression of mitochondrial biogenesis marker proteins; particularly, COX-IV exhibited a higher plasticity related to SDH in response to HIIT in the soleus and rectus femoris. Consistently, a recent comparison of HIIT and MICT also demonstrated that interval training compared with volume-matched continuous single-leg cycling elicited superior mitochondrial adaptations in human skeletal muscle, as indicated by higher COX-IV content in the rectus femoris after training (MacInnis et al., 2017). Our findings are in agreement with those of previous studies that have demonstrated elevation of SDH activities in the oxidative soleus and gastrocnemius did not differ between exercise modalities (Criswell et al., 1993). Our data also showed that HIIT was more effective in increasing the expression of SDH and COX-IV proteins, which improved cardiac efficiency by increasing myocardial glucose oxidation with concomitant maximal mitochondrial respiratory capacity of the myocardium, when compared with MICT (Hafstad et al., 2011). These data are similar to those of Kainulainen et al. (1990) in hearts from exercise-trained rats, which showed similarly unaltered SDH and COX-IV activity after a speed-increased (running 1 h at an increased speed to 20 m min^−1^) and speed-constant (running 1 h at a constant speed of 25 m min^−1^) 5-weeks endurance exercise regimen. These results, together with those presented herein, demonstrate that the myocardium exhibits less metabolic plasticity in response to MICT than skeletal muscles, but HIIT facilitates cardiac adaptations that increase mitochondrial oxidative capacity by increasing the expression of SDH and COX-IV proteins.

Mitochondrial adaptations are proposed to be mediated in part by SIRT3, which, as an important regulator of mitochondrial function and/or biogenesis, deacetylates many mitochondrial enzymes to orchestrate metabolic alteration (Vassilopoulos et al., 2014) and regulates expression of proteins, such as SDH, in the mitochondrial electron transport chain (Finley et al., 2011). The transgenic mouse model with muscle-specific expression of SIRT3 exhibited better exercise tolerance on treadmills related to mitochondrial oxidative capacity and mitochondrial biogenesis in skeletal muscle (Lin et al., 2014). Following this, several studies reported that exercise training or electrical stimulation (Gurd et al., 2012; Hokari et al., 2012; Edgett et al., 2016) increased skeletal muscle SIRT3 expression and was specific to muscles recruited during exercise intervention. Studies in rodent models have shown that SIRT3 expression increased in the rectus femoris and oxidative soleus muscle following endurance training (Hokari et al., 2012). Consistently, we observed significance SIRT3 expression increase in rectus femoris (Figure 1B) and oxidative soleus muscle (Figure 1A), but with no concomitant changes in cardiac muscle in both exercise modalities (Figure 1C), suggesting that the exercise-induced increase in SIRT3 expression was strongly dependent on muscle type. This finding is in agreement with treadmill running- and voluntary running training-induced elevation of SIRT3 protein expression in vastus lateralis, soleus, and triceps muscle (Hokari et al., 2012). The discrepancy between the findings of this study and those of Edgett et al. (2016) and Casuso et al. (2017), which showed no change in SIRT3 content in the vastus lateralis following 3 or 6 weeks of sprint-interval training, may be explained by differences in training protocol. Indeed, our data also showed HIIT was superior to volume-matched MICT in enhancing SIRT3 expression in skeletal muscle (Figures 1A,B), suggesting regulation of SIRT3 expression depends strongly on training protocol. In addition, our data showed no change in SIRT3 content in cardiac muscle, which is inconsistent with previous findings of upregulation of cardiac muscle SIRT3 expression in a rodent model of myocardial infarction following HIIT (Jiang et al., 2014). It is possible that SIRT3 expression in cardiac muscle in the myocardial infarction model is more sensitive to changes in response to HIIT. The upregulation of SIRT3 correlated with enhanced downstream SDH and COX-IV content in rectus femoris and soleus muscle (Table 3), suggesting that SIRT3 expression may be required for exercise-induced increases of mitochondrial oxidative capacity in skeletal muscle. Meanwhile, HIIT resulted in greater expression levels of SIRT3 with concomitant changes in COX-IV content in skeletal muscle than those in the MICT group (Figure 1B). We thus hypothesized that SIRT3 rendered HIIT superior to MICT by larger improvement of exercise tolerance and mitochondrial oxidative capacity in skeletal muscle.

Two recent studies suggested that mitochondrial ALDH2, an upstream signaling molecule regulating SIRT3 expression, in the cardiac and skeletal muscle could participate in the detoxification of acetaldehyde (Zhang et al., 2017) and be involved in the regulation of oxidative stress associated with facilitation of SIRT3-dependent PGC-1α deacetylation (Hu et al., 2016). A recent study implied that skeletal muscle-specific ALDH2 overexpression restored exhaustive exercise-induced mitochondrial dysfunction in skeletal muscle through maintaining mitochondrial function in mitochondrial complexes I and V. The benefits of ALDH2 activation include protection against various heart diseases, induced cardiomyocytes, mitochondrial injuries, and apoptosis (Zhang et al., 2017). Data from our current study showed that HIIT significantly increased ALDH2 in the rectus femoris muscle but with no concomitant changes in oxidative soleus and cardiac muscle (Figures 1A–C). Moreover, we found that upregulation of ALDH2 correlated with COX-IV, Beclin-1, and SIRT3 content in rectus femoris muscle (Table 3). However, whether ALDH2 content in rectus femoris muscle takes part in mechanistic events contributing to SIRT3 improvement of mitochondrial function induced by HIIT needs to be addressed in future studies. Additionally, the previous study also reported no changes in ALDH2 expression in cardiac muscle of hypertensive rats following endurance training (Campos et al., 2015). Thus, additional studies are needed to determine effects of exercise intensity, duration, and volume on ALDH2 expression in cardiac and skeletal muscles in different training models.

### Basal autophagic activity

Exercise is known to be implicated in the regulation of basal autophagic activity in a tissue-dependent manner. Endurance training induced a significant increase in autophagy markers, such as Beclin-1, LC3-II, and p62, in cerebral tissues and quadriceps but not liver, gastrocnemius, and cardiac muscle in rats (Bayod et al., 2014). Our study compared basal autophagic activities in the oxidative soleus, glycolytic rectus femoris, and left ventricular muscle following 10-weeks HIIT and MICT programs (Figures 1D–F). Compared with oxidative soleus, the cardiac muscle continually contracts and has the highest aerobic capacity and metabolic demand of all muscles (Park et al., 2014). The present findings, which showed no significant change in LC3-II, LC3-II/LC3-I ratio, ATG-3, and Beclin-1 expression in the left ventricular, glycolytic rectus femoris, and oxidative soleus muscle of rats in the MICT group compared to those in the SED group (Figures 1D,F), suggested that the basal autophagic activities remained stable among these muscles after 10-weeks MICT. This observation supports previous studies reporting no changes in the oxidative soleus (Lira et al., 2013; Tam et al., 2015) and cardiac muscle (Haram et al., 2009; Smuder et al., 2013; McMillan et al., 2015) following long-term voluntary running and MICT. In contrast, a previous study has shown that MICT increased basal autophagic flux and expression of autophagy proteins in parallel to mitochondrial biogenesis only in the plantaris muscle with mixed fiber types (Schwalm et al., 2015), which implies exercise-induced increase in mitochondrial function would require high basal autophagic activity-meditated mitochondrial turnover (Lira et al., 2013). Here, we speculated that improvement of mitochondrial function following MICT may be achieved only by biosynthesis of new mitochondria and not basal autophagic activity-meditated removal of damaged mitochondria. Moreover, the study of McMillan et al. (2015) agreed with that of Sun et al. (2013), who reported that increasing the volume and duration of exercise (more than in our study) and matching intensity increased autophagy flux, including elevated LC3-II and Beclin-1 expression and LC3-II/LC3-I ratio, inferring that alteration of basal autophagic activities seems to rely on training volume.

To our knowledge, this is the first study to investigate the effects of HIIT on autophagy marker content in the cardiac, glycolytic rectus femoris, and oxidative soleus muscle of rats. Our observations in the soleus and cardiac muscle showed that, compared with those in the SED group, HIIT can increase LC3-II protein expression as well as the LC3-II/LC3-I ratio, which is the most commonly used marker in monitoring autophagic flux (Figures 1D,F). We also measured autophagic activities using other markers that promote the initial assembly of the autophagosomal membrane, including ATG-3 and Beclin-1, which are involved in the upregulation of autophagosome synthesis and formation of the first ubiquitin-like conjugation system, indicating that ATG-3 and Beclin-1 expression increased in oxidative soleus and cardiac muscle after HIIT compared with the values in the SED group (Figures 1D,F). Our data showed that the LC3-II/LC3-I ratio had significant positive relationships with the SDH content in soleus and cardiac muscle (Table 3), suggesting that elevation of basal autophagy flux induced by HIIT in soleus and cardiac muscle contributes to improved mitochondrial oxidative capacity. Importantly, SIRT3 content was positively correlated with the LC3-II/LC3-I ratio in soleus muscle (Table 3); we thus hypothesize that SIRT3 mediates the upregulation of basal autophagic flux in oxidative soleus muscle through an unexplored transcription-dependent mechanism.

### Metabolomics

Exercise training effectively enhances the rates of energy expenditure and substrate flux, creating an ideal situation for large-scale metabolomic profiling. Additionally, plasma metabolites of exercise were found to be correlated with fitness in healthy individuals, reflecting underlying glucose utilization and lipid metabolism (Lewis et al., 2017). As we can see in Figure 2, PCA and PLS-DA analysis of serum samples showed SED group rats were clustered far away from HIIT or MICT group rats, indicating that there were notable metabolic changes induced by both exercise modalities. Others have similarly showed clear separation of the SED group from the groups involving continuous and high-intensity interval aerobic exercise by well-trained male cyclists and triathletes (Peake et al., 2014). However, when assessing the differential changes between programs of exercise in the rat serum metabolomic profile, we found that the MICT group overlapped with the HIIT group (Figures 2B,D), showing that the metabolic change was small between the two training groups. Furthermore, when evaluating the metabolite profile alterations of two types of exercise training, 11 metabolites with higher VIP scores were selected as the most important in groups segregated in the PLS-DA model shown in Figure 2C. Interestingly, five metabolites, including glucose, lysine, choline, 3-hydroxybutyrate, and tyrosine, showed a similar change in both exercise modalities, while BCAAs and glutamine specifically increased in the HIIT group when compared with those in the SED group.

Our study showed an increase in the level of tyrosine after 10-weeks HIIT and MICT programs (Table 4). As an aromatic amino acid, tyrosine is a precursor of neurotransmitters and is converted into the catecholamine hormones norepinephrine and epinephrine, both released by the adrenal glands in response to stress, including exercise training (Erdem et al., 2002). These findings were in agreement with studies in male C57BL/6J mice from Duggan et al. (2011) and Monleon et al. (2014), who have shown that short-term MICT and long-term spontaneous exercise caused a higher concentration of tyrosine. Additional metabolites that increased with both exercise modalities were choline, 3-hydroxybutyrate, and lysine, which are implicated in the regulation of lipid homeostasis (Penry and Manore, 2008). It is suggested that supplementation with choline augments exercise performance through promotion of fatty acid oxidation and enhanced utilization of fat as an energy substrate. 3-hydroxybutyrate is a carrier of energy from liver to peripheral tissues during exercise in animals (Sachan and Hongu, 2000) and humans (Hongu and Sachan, 2003). Lysine can provide the carbon backbone for carnitine, a critical component in the transportation of fatty acids into the mitochondrial matrix to yield energy through β-oxidation. Higher concentrations of these three metabolites in exercising animals are indicative of a greater reliance on fatty acid oxidation as an energy substrate.

Previous metabolomic studies have noted increases in plasma glutamine release from skeletal muscle after long-term strength and endurance training (Rowbottom et al., 1997). Exercise training has been shown to increase glucose transporter (GLUT4) expression and glucose tissue utilization in this model (Fueger et al., 2004). Our data was consistent with another previously reported study using metabolomics, in which lowered glucose levels were found in mice doing long-term wheel running (Monleon et al., 2014). Although we did not measure GLUT4 content, muscle glycogen synthesis, and insulin sensitivity in skeletal and cardiac muscle, it has been previously reported that an increase in muscle glycogen synthesis via higher insulin sensitivity and resting total GLUT4 protein in skeletal muscle after exercise lowered glucose levels (Monleon et al., 2014).

An additional metabolite specifically elevated after HIIT was glutamine. Previous metabolomic studies have noted increases in plasma glutamine release from skeletal muscle after long-term strength and endurance training (Rowbottom et al., 1997). There was also evidence to suggest that the single measure of an elevated plasma glutamine concentration in an athlete represents a positive adaptation to a well-balanced training program (Rowbottom et al., 1995) and is often used as an indicator of tolerance to volume of work (Smith and Norris, 2000).

Most notably, our study showed that the level of BCAAs valine, isoleucine, and leucine was specifically elevated in the HIIT but not MICT group, when compared with those of the SED group (Table 3). There was evidence that efficient BCAA utilization contributed to high intrinsic exercise capacity and enhanced fitness (Overmyer et al., 2015), which is associated with activation of fatty acid oxidation and promotion of muscle-protein synthesis during submaximal exercise (Shimomura et al., 2004). A previous study in C57BL/6J mouse has shown that 6-weeks HIIT resulted in a significant attenuation of the exercise-induced increase in BCAA oxidation, with a concomitant attenuation of branched-chain 2-oxoacid dehydrogenase (BCOAD, associated with a reduced rate of whole-body BCAA oxidation) percent activation (Howarth et al., 2007), which was related to an increased muscle oxidative capacity and reduced cellular energy disturbance (McKenzie et al., 2000). Although speculative, HIIT-induced elevation of BCAAs, as shown in the present study, may be responsible for attenuation of the exercise-induced increase in BCAA oxidation by reducing BCOAD activity, and this was associated with larger improvement of exercise tolerance and mitochondrial oxidative capacity in skeletal muscle after the 10-weeks HIIT program. Thus, it seems that differences in BCAA metabolism in HIIT relative to MICT programs may be attributed to high intrinsic exercise capacity and beneficial mitochondrial adaptations in skeletal and cardiac muscle promoted by exercise training.

### Limitations

There are several limitations of this study worth discussing. First, the finding that the positive adaptations to HIIT were superior to those of volume-matched MICT should be further tested in humans. Indeed, a growing body of evidence suggests that HIIT is a potent and time-efficient strategy to induce metabolic adaptations and improve exercise capacity (Little et al., 2010). Given that ‘lack of time’ is the most commonly cited barrier to performing regular exercise in a variety of populations, HIIT may represent an alternative to endurance training to improve metabolic health (Trost et al., 2002). Second, limitations of the present study included the measurement of metabolites by NMR. This technology is not as sensitive as mass spectroscopy and detects only metabolites at higher concentrations within the metabolome. Third, the small sample size for the protein expression and plasma metabolomic profile measurements was also a limitation. Another potential limitation included the measurement of serum and not skeletal and cardiac muscle; we cannot pinpoint the source or tissue responsible for these differences, because the plasma metabolomic footprints are global and do not provide tissue-specific metabolic fingerprints. Additionally, we only reported data for all variables following intervention; there is obviously no control for the basal state. Lastly, the exercise sessions of the HIIT and MICT groups were performed with identical frequency and were time-matched, with both training sessions lasting 40 min, and calculated cumulative work performed each week was comparable between HIIT and MICT.

## Conclusions

In summary, HIIT was more effective for improving physical performance and facilitating cardiac and skeletal muscle adaptations that increase mitochondrial oxidative capacity and basal autophagic activities than a volume-matched MICT protocol. Meanwhile, our findings highlight the benefits of untargeted metabolomic profiling of plasma from rat after HIIT and MICT programs to characterize similar adaptive changes in the metabolome through detection of metabolites glucose, lactate, lysine, choline, glutamate, 3-hydroxybutyrate, and tyrosine. Of note, our results also showed that BCAAs and glutamine were specifically elevated by HIIT, suggesting that BCAAs and glutamine may represent a positive adaptation to HIIT.

## Author contributions

F-HL, TL, and J-YA have contributed equally to this work. F-HL, TL, and J-YA conceived and designed the experiments and contributed to the writing and editing of the manuscript. F-HL and TL performed the experiments. F-HL, LS, LZ and TL analyzed the data. F-HL, TL, YL, ZM, RD, J-YA, and TC-YL contributed to discussions and provided required reagents, materials, analysis tools.

### Conflict of interest statement

The authors declare that the research was conducted in the absence of any commercial or financial relationships that could be construed as a potential conflict of interest. The reviewer DRJ and handling Editor declared their shared affiliation.

## Abbreviations

^1^H NMR: proton nuclear magnetic resonance
ANOVA: analysis of variance
BCAAs: branch-chained amino acids
BW: body weight
CI: confidence intervals
ES: effect sizes
FDR: false discovery rate
HIIT: high-intensity interval training
MICT: moderate-intensity continuous training
PCA: principle component analysis
PLS-DA: partial least squares discriminant analysis
SED: sedentary control
VIP: variable importance for projection
VO_2max_: maximal oxygen uptake
